# Outcomes in Lung Cancer: 9-Year Experience From a Tertiary Cancer Center in India

**DOI:** 10.1200/JGO.2016.006676

**Published:** 2017-01-11

**Authors:** Aditya Navile Murali, Venkatraman Radhakrishnan, Trivadi S. Ganesan, Rejiv Rajendranath, Prasanth Ganesan, Ganesarajah Selvaluxmy, Rajaraman Swaminathan, Shirley Sundersingh, Arvind Krishnamurthy, Tenali Gnana Sagar

**Affiliations:** All authors: Cancer Institute (WIA), Adyar, Chennai, India.

## Abstract

**Purpose:**

Lung cancer is the most common cause of cancer mortality in the world. There are limited studies on survival outcomes of lung cancer in developing countries such as India. This study analyzed the outcomes of patients with lung cancer who underwent treatment at Cancer Institute (WIA), Chennai, India, between 2006 and 2015 to determine survival outcomes and identify prognostic factors.

**Patients and Methods:**

In all, 678 patients with lung cancer underwent treatment. Median age was 58 years, and 91% of patients had non–small-cell lung cancer (NSCLC). Testing for epidermal growth factor receptor mutation was performed in 132 of 347 patients and 61 (46%) were positive.

**Results:**

Median progression-free survival was 6.9 months and overall survival (OS) was 7.6 months for patients with NSCLC. Median progression-free survival was 6 months and OS was 7.2 months for patients with small-cell lung cancer. On multivariable analysis, the factors found to be significantly associated with inferior OS in NSCLC included nonadenocarcinoma histology, performance status more than 2, and stage. In small-cell lung cancer, younger age and earlier stage at presentation showed significantly better survival.

**Conclusion:**

Our study highlights the challenges faced in treating lung cancer in India. Although median survival in advanced-stage lung cancer is still poor, strategies such as personalized medicine and use of second-line and maintenance chemotherapy may significantly improve the survival in patients with advanced-stage lung cancer in developing countries.

## INTRODUCTION

Lung cancer is one of the most common causes of cancer-related deaths worldwide. In India, lung cancer accounts for 9.3% of all cancer-related deaths in both sexes.^1^ There are few studies on survival outcomes of lung cancer in India. This study analyzed outcomes in patients with lung cancer treated at our center and identified prognostic factors.

## PATIENTS AND METHODS

Clinical and treatment details of all consecutive patients with lung cancer who underwent treatment at our center between January 2006 and June 2015 were collected and analyzed retrospectively. The study was approved by the Institute Ethics Committee. Never-smokers were defined as those who had smoked fewer than 100 cigarettes during their lifetime; ever smokers were defined as those who had smoked 100 cigarettes or more during their lifetime.^2^ Diagnosis was established by core needle biopsy or fine-needle aspiration cytology. Histopathologic examination and immunohistochemistry were performed to classify non–small-cell lung cancer (NSCLC) and small-cell lung cancer (SCLC). NSCLC was further categorized as adenocarcinoma, squamous cell carcinoma, or poorly differentiated carcinoma. Molecular testing for epidermal growth factor receptor (EGFR) mutation was begun in our center in 2012, and testing for anaplastic lymphoma kinase (ALK) translocation was begun in 2014. EGFR mutation analysis was performed by using reverse transcriptase polymerase chain reaction, and ALK translocation analysis was performed by immunohistochemistry using an anti-ALK rabbit monoclonal antibody (Ventana Medical Systems, Tucson, AZ).^3^ Molecular testing was performed only in patients who had adequate tissue material for analysis. For staging, patients underwent contrast-enhanced computed tomography (CT) scanning of the chest, ultrasound scan of the abdomen and pelvis, and bone scan or whole-body positron emission tomography (PET)/CT scanning. In addition, patients with SCLC underwent bone marrow trephine biopsy.

Treatment was based on physician discretion and patient choice. Responses were assessed by using chest x-ray, contrast-enhanced CT, or PET/CT after four cycles of intravenous chemotherapy. Assessments were performed at the physician’s discretion between 3 and 6 months after the start of tyrosine kinase inhibitors (TKIs) and repeated again at the same intervals. Radiologic assessment was also performed if the patient had signs and symptoms of disease progression at any time during follow-up. Response Evaluation Criteria in Solid Tumors (RECIST) was used for assessment of response.^4^ Patients who were started on treatment and were followed up for at least one visit or those whose outcome was known were included in the survival analysis. Patients who were started on treatment and died before response assessment were also included. Patients who did not complete the first follow-up visit and those whose survival status was not known were not included.

Progression-free survival (PFS) was calculated from date of initiation of treatment to date of disease progression or relapse. Overall survival (OS) was calculated from date of initiation of treatment to date of last follow-up or date of death. For survival analysis, all patients were censored at date of last follow-up or date of contact by telephone or mail if they were lost to follow-up or on December 31, 2015, whichever was earlier. PFS and OS were analyzed by the Kaplan-Meier method, and risk factors were compared by using the log-rank test for univariable analysis and a Cox proportional hazards model for multivariable analysis. SPSS version 17.0 (SPSS, Chicago, IL) was used for statistical analysis.

## RESULTS

During the study period, 1,039 patients were registered in the hospital with a diagnosis of lung cancer. Records were available for 1,006 patients. Of these, 866 patients had histologically proven lung cancer. One hundred twenty-six of 866 patients did not receive any treatment because of either poor performance status (PS) or patient preference. Sixty-two patients began treatment but had no follow-up details and thus were excluded from the survival analysis. A total of 678 patients underwent treatment for lung cancer and had at least one follow-up visit; they were included in the outcome analysis.

### Baseline Characteristics

The median age was 58 years (range, 20 to 83 years). Males constituted 516 (76%) of 678 patients. Cough was the most common presenting complaint seen in 302 (44.5%) of 678 patients. Lung cancer was incidentally detected in nine (1.3%) of 678 patients (Table 1). History of smoking was present in 362 (53.4%) of 678 patients, and the median number of pack-years was 20 (range, 3 to 80 pack-years). Thirty (4.5%) of 678 patients had a previous history of tuberculosis. The majority of the patients had a PS of 1 or 2.

**Table 1:**
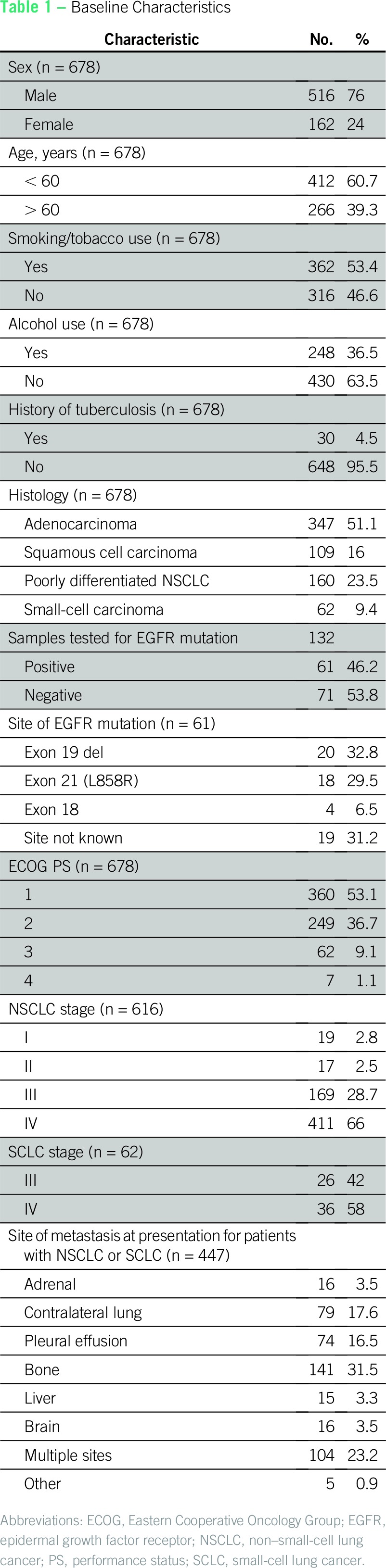
Baseline Characteristics

### Diagnosis and Pathology

Diagnosis of lung cancer was confirmed by using excision or guided core needle biopsy in 497 (74%) of 678 patients or by using fine-needle aspiration cytology in 181 (26%) of 678 patients. NSCLC was diagnosed in 616 (91%) of 678 patients and SCLC was diagnosed in 62 (9%) of 678 patients. The most common histologic subtype among patients with NSCLC was adenocarcinoma, which was recorded in 347 (56.3%) of 616 patients followed by squamous cell carcinoma in 109 (17.7%) of 616 patients. A total of 132 samples were tested for EGFR mutation, of which 61 (46.2%) were positive. Exon 19 was the most common type of EGFR mutation and was observed in 20 (33%) of 61 patients. Exact type of EGFR mutation was not known in 19 patients. Testing for ALK mutation was performed in 32 patients with adenocarcinoma of whom two (6.25%) were found to be positive.

### Staging and Treatment

#### NSCLC.

NSCLC was diagnosed in 616 (91%) of 678 patients. The majority of patients presented with disseminated disease: 411 (67%) of 616 were stage IV, and the most common site of metastasis was bone in 126 (31%) of 411. Ninety-seven patients (23.2%) had more than one site of metastasis.

Thirty-six patients were either stage I or II at presentation. Of these, two patients received oral gefitinib empirically because they were not eligible for surgery or radiotherapy. Of the remaining 34 patients, 19 underwent only surgical resection, nine received adjuvant chemotherapy after surgical resection, two received neoadjuvant chemotherapy followed by surgery, and four received definitive radiotherapy only. Among 169 patients with stage III disease, 12 (7%) of 169 received oral TKIs, and two (1%) of 169 underwent surgery followed by adjuvant chemotherapy. Thirty-six (21%) of 169 patients received concurrent chemotherapy and radiotherapy, and 39 (23%) of 169 received chemotherapy followed by sequential radiotherapy. The remaining 80 patients received either intravenous or oral chemotherapy only. Among 411 patients with stage IV disease, 169 (41%) of 411 received intravenous chemotherapy, and 179 (43.5%) of 411 received oral EGFR TKIs such as gefitinib or erlotinib as first-line therapy. Sixty-one patients received only oral etoposide because they were deemed ineligible for intravenous chemotherapy, and two received only radiotherapy for the same reason. Among patients with metastatic disease who received intravenous chemotherapy, 70 (42%) of 169 received gemcitabine and platinum doublet. Fifty-eight (35%) of 169 patients received pemetrexed and platinum doublet, which was used mostly after 2012 when generic medications became available. Twenty-six patients received maintenance pemetrexed chemotherapy after completing four or six cycles of initial chemotherapy.

#### SCLC.

Sixty-two patients (9%) were diagnosed with SCLC, and 51 (82%) of 62 were ever smokers. The majority of patients presented with disseminated disease: 36 (58%) of 62 were stage IV, and the most common site of metastasis at presentation was bone in 15 (42%) of 36 patients. Seven patients (19%) had more than one site of metastasis.

Among 26 patients with stage III disease, one patient received intravenous chemotherapy only, and three patients received only oral etoposide because of poor general condition. Thirteen (50%) of 26 patients received concurrent chemotherapy and radiotherapy, and nine (35%) of 26 received chemotherapy followed by sequential radiation. Among 36 patients with stage IV disease, 22 (61%) of 36 received intravenous chemotherapy and 14 (39%) of 36 received oral etoposide only because they were deemed ineligible for intravenous chemotherapy. Among patients with metastatic disease who received intravenous chemotherapy, 14 (67%) of 22 received cisplatin and etoposide doublet and eight (23%) of 22 received carboplatin and etoposide doublet.

### Survival Outcomes in NSCLC

**Fig 1:**
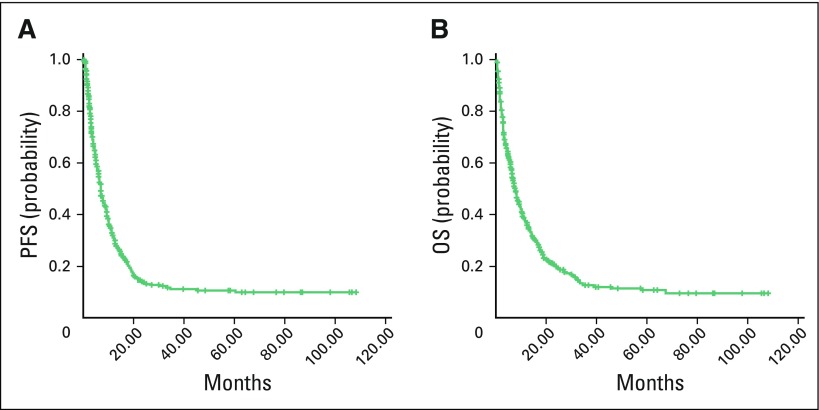
Kaplan-Meier estimates of (A) progression-free survival (PFS) and (B) overall survival (OS) in non–small-cell lung cancer for all stages.

Of the 678 patients with lung cancer in our study, 616 were found to have NSCLC, and survival outcomes were analyzed for that group. Median duration of follow-up was 6.3 months (range, 0.1 to 108.1 months). Median PFS for patients with all stages of NSCLC was 6.9 months, and median OS was 7.6 months (Fig 1). Survival status was not known for 91 patients because they could not be contacted by telephone or mail. Thus, they were censored to be alive for survival analysis. On univariable analysis, smoking, alcohol consumption, histologic subtype of NSCLC, PS, disease stage, and sex were significant predictors of OS (Table 2). On multivariable analysis, histology, stage, and PS were significant predictors of OS (Data Supplement). Disease stage and PS were significant predictors of PFS on univariable analysis (Table 2) and multivariable analysis.

**Table 2:**
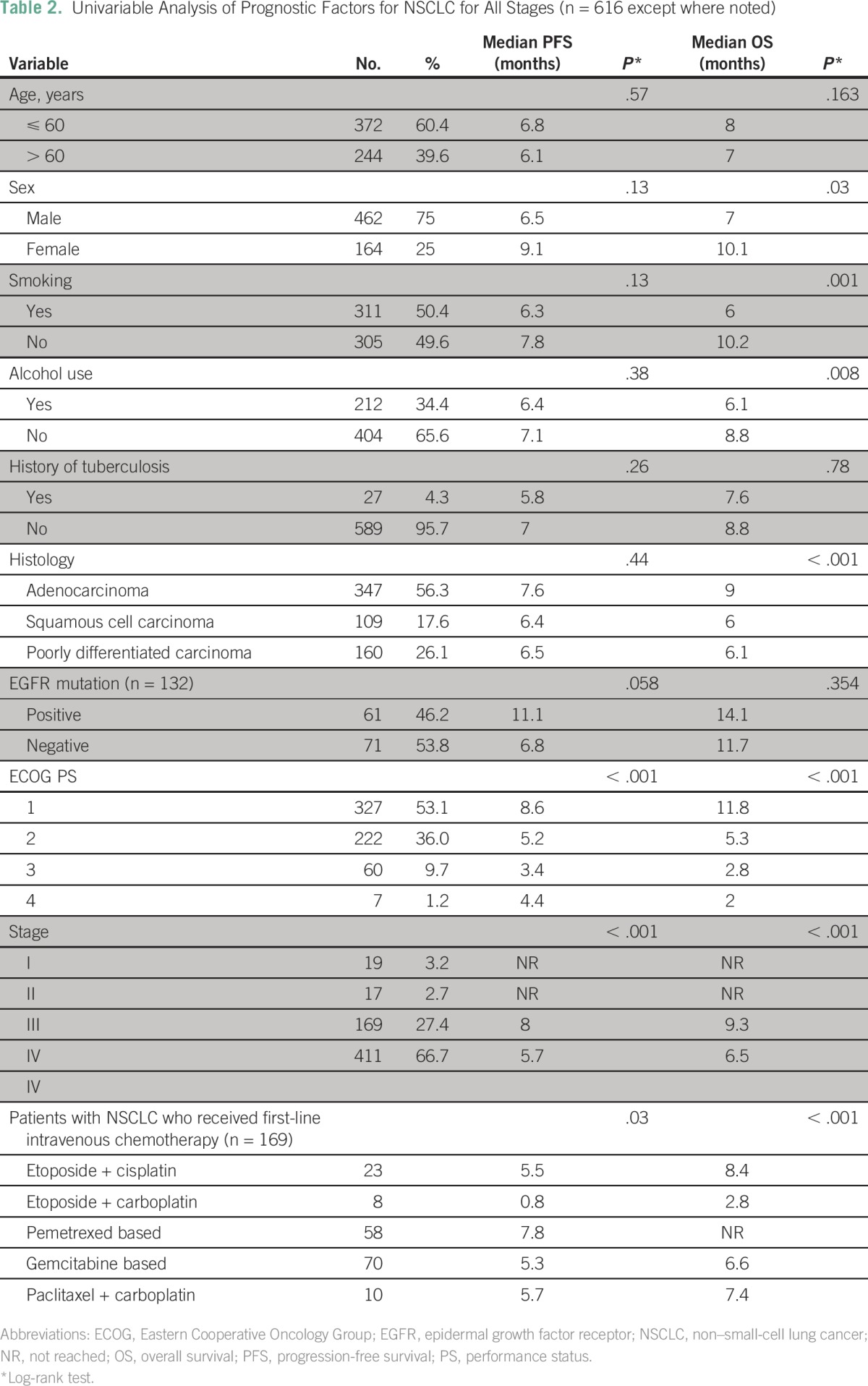
Univariable Analysis of Prognostic Factors for NSCLC for All Stages (n = 616 except where noted)

The 1-year survival for patients with stage I disease (n = 19) was 83%, and it was 76% for those with stage II disease (n = 17; Data Supplement). Patients with stage IV disease (n = 411) had a median PFS of 5.7 months and a median OS of 6.5 months (*P* < .001). In patients with stage IV NSCLC who received first-line intravenous chemotherapy, the median OS had not yet been reached in those who received pemetrexed combinations (n = 58). Patients with stage III NSCLC who received concurrent chemotherapy and radiotherapy (n = 36) had better PFS (31% *v* 8%; *P* = .29) and OS (30% *v* 0%; *P* = .51) compared with those who received chemotherapy followed by sequential radiotherapy (n = 39). Patients with stage IV NSCLC who received maintenance chemotherapy (n = 26) had a median PFS of 9.6 months and a median OS of 24.9 months.

Second-line treatment was given to 107 (26%) of 411 patients with stage IV disease on progression, among whom 63 of 107 received intravenous chemotherapy and 44 of 107 received oral TKIs. The median PFS was 3.6 months for second-line treatment, 4.1 months for oral TKIs, and 3.46 months for intravenous chemotherapy.

### Survival Outcomes in SCLC

**Fig 2:**
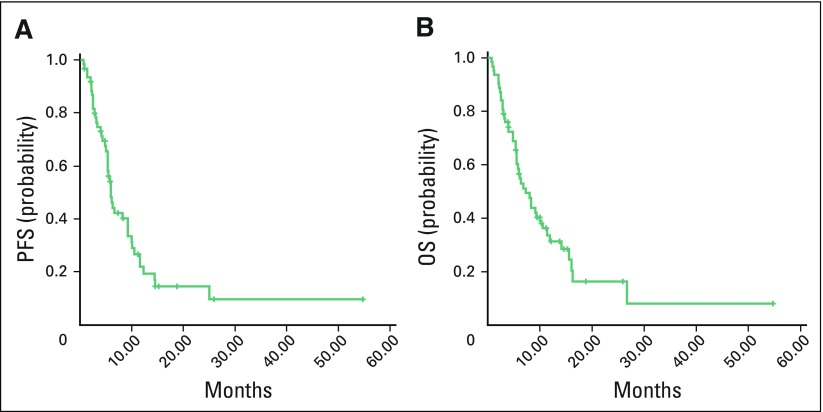
Kaplan-Meier estimates of (A) progression-free survival (PFS) and (B) overall survival (OS) in small-cell lung cancer for all stages.

Sixty-two of 678 patients with lung cancer were found to have SCLC. Median duration of follow-up was 6.1 months (range, 0.7 to 54 months). Median PFS for all stages was 6.0 months, and median OS for all stages was 7.2 months (Fig 2; Table 3).

**Table 3:**
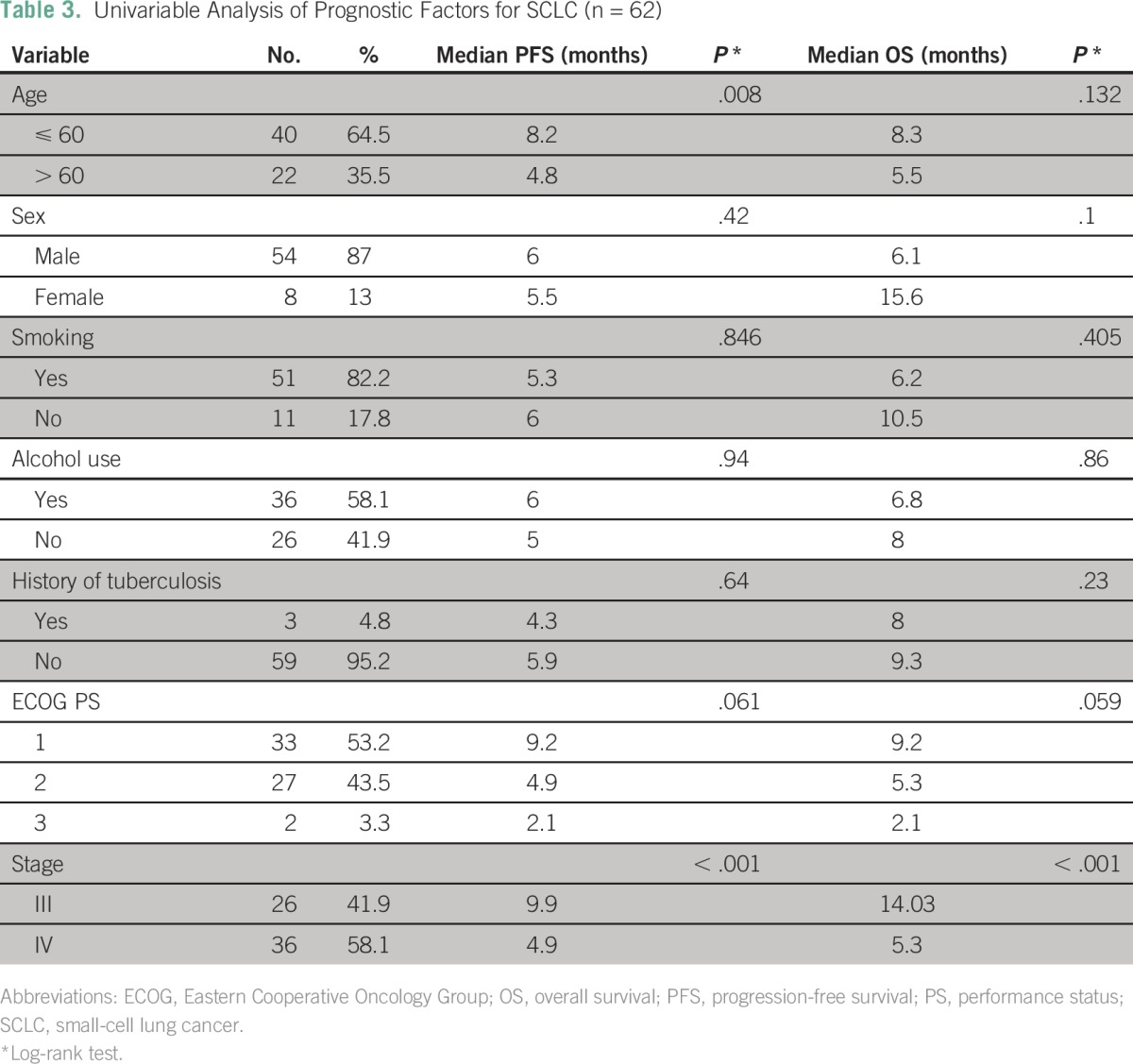
Univariable Analysis of Prognostic Factors for SCLC (n = 62)

Age and stage at presentation were the only two factors that were significantly associated with survival in patients with SCLC (Table 3). Patients with SCLC who presented with stage III disease and received concurrent chemotherapy and radiotherapy had better PFS (46.2% *v* 33.3%; *P* = .53) and OS (24% *v* 0%; *P* = .43) compared with those who received sequential chemotherapy and radiotherapy, but it was not statistically significant. Second-line chemotherapy was given to 16 of 62 patients, and the median PFS was 2.9 months.

## DISCUSSION

Treatment of advanced lung cancer in India is accompanied by a unique set of challenges. Access to quality oncologic care is limited because of the scarcity of resources and qualified professionals. The cost of mutation analysis for patients with lung cancer in India is still substantial.^5^ With the advent of generic drugs, the cost of EGFR TKIs has substantially decreased making them affordable for a wider patient population. However, ALK inhibitors are still prohibitively expensive and are expected to remain so for the foreseeable future. There are only a few studies in India that have reported outcome data in patients with advanced-stage NSCLC (Table 4).

**Table 4:**
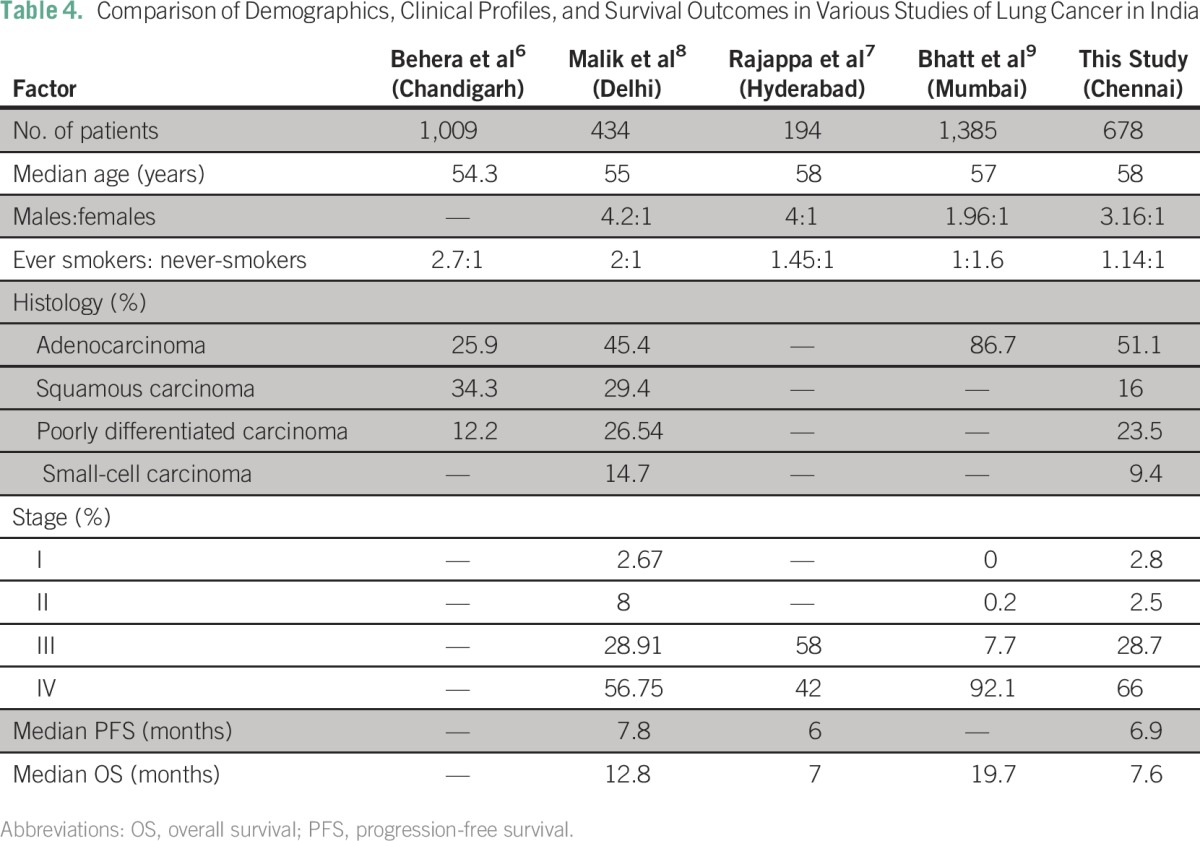
Comparison of Demographics, Clinical Profiles, and Survival Outcomes in Various Studies of Lung Cancer in India

In our study, the median age of presentation was 58 years, which is a decade younger compared with the European population, but it is comparable to that in other reports from India.^7-10^ The majority of patients (53.4%) in our study were smokers. This is a lower rate than in other studies in North India but is comparable to that in other studies in South India.^7,8^ This may reflect the variations in tobacco consumption practices among different geographic regions in India. In our study, 76% of the patients were males which may be a result of decreased smoking habits among women. This is lower compared with the distribution of the sexes in other international studies,^10,11^ but it is comparable to data from other Indian centers.^7,8^

The majority of the patients in our study had adenocarcinoma histology (51%). This is consistent with other Indian studies in the current era and indicates a shift from predominantly squamous histology as was observed earlier and may reflect changes in smoking practices and the increasing use of filtered cigarettes.^7,8,12^ Most of our patients presented with advanced lung cancer with 66% having stage IV disease at diagnosis. However, this is comparable to other Indian studies and indicates a delay in seeking treatment.^8^

Most of the patients in this study (89.7%) had a PS of 1 or 2. This was higher than what has been observed in other Indian studies,^7,8^ probably because our study included only those patients who received some form of treatment. Patients with poor PS were referred for best supportive care at a nearby palliative care center. However, none of our patients had a PS of 0 at presentation.

Evaluation of tumors for EGFR mutation was started in 2012; at that time, 132 patients were examined and 46% of tumors were positive for an EGFR mutation. This is higher than in other studies performed in India by Doval et al (33%)^13^ and Bhatt et al (31.3%),^9^ possibly as a result of selection bias because the testing was aimed more at female patients and never-smokers. In the study titled “First Line IRESSA Versus Carboplatin/Paclitaxel in Asia (IPASS)” in East Asia in which only a selected population of nonsmokers with adenocarcinoma of the lung were included, EGFR mutations were present in 59.7%.^14^ In our study, the most common sites of EGFR mutation were deletion in exon 19 and mutation at exon 21 (L858R). Together, these accounted for 62% of the mutations. These two mutations were also the predominant mutations in the IPASS study.^14^ Approximately 6% of the patients in our study tested positive for ALK translocation. This is higher compared with results from other centers (2.7% to 3%) in India.^13,15^ This might be a result of the use of immunohistochemstry rather than fluorescent in situ hybridization for detecting translocations.^16^

Although the survival outcomes in this study are similar to those in another study from South India, they are inferior to the outcomes from other studies in North India.^7-9^ The median PFS was 6.9 months for patients with NSCLC in our study and 7.8 months in the study by Malik et al.^8^ We analyzed the patients in our study from date of treatment initiation, unlike Malik et al who analyzed patients from date of presentation to the hospital; assessment of response was also performed earlier in our study compared with theirs. These factors could account for the improvement of 1 month in PFS in their study. Our median OS was 7.6 months, whereas Malik et al reported a median OS of 12.8 months; however, the majority of our patients with stage IV disease (74%) did not receive second-line chemotherapy. Details about second-line or maintenance treatment were not available in the article by Malik et al. Bhatt et al^9^ did not report on median PFS, second-line treatment, or response assessment in their study.

In patients who had EGFR-positive stage IV NSCLC, the median PFS was 11.1 months and median OS was 14.1 months, which is comparable to those in other studies performed worldwide.^10,11^ Patients who had EGFR-negative stage IV NSCLC had a significantly better PFS and OS with pemetrexed-based chemotherapy regimens. The combination of cisplatin and etoposide showed a better OS (8.4 months) compared with all other regimens; thus, use of this regimen is still a reasonable option in a resource-challenged setting.

Concurrent chemotherapy and radiotherapy was associated with better OS compared with chemotherapy followed by sequential radiation in both SCLC and NSCLC, although this was not statistically significant. Thus, in all eligible patients, concurrent chemotherapy and radiotherapy should be considered as the treatment modality of choice.

Use of maintenance pemetrexed in stage IV NSCLC led to a better OS, which demonstrates the role of maintenance chemotherapy in stage IV NSCLC. In our study, patients who received maintenance chemotherapy had a median PFS of 9.6 months and median OS of 24.9 months. In a study by Pandey et al^17^ of maintenance pemetrexed for 188 patients, the median PFS was 8 months and median OS was 20 months. However, the retrospective nature of this study precludes us from making conclusions.

There is a paucity of data on SCLC in India. In SCLC, on univariable analysis, only stage of disease was shown to significantly affect OS in this study. The study by Malik et al^8^ included 64 patients with SCLC. Median PFS (6.8 *v* 6 months) and OS (9.1 *v* 7.2 months) were higher compared with those in this study.

Our results are limited by their retrospective nature and the fact that widely heterogeneous treatment modalities were used over a period of 9 years. Mutation testing was not performed in many patients for economic reasons. The final survival analysis did not include 62 of 866 patients who received treatment but did not have any follow-up and 126 of 866 patients who did not receive any treatment because of poor PS or patient preference. About 14% of patients were lost to follow-up (91 of 616). The above issues related to data are confounding factors in our analysis and may have a significant impact on projected survival for patients with lung cancer in India. However, unlike controlled conditions in a trial, these data reflect circumstances in the field.

Lung cancer is a common cause of cancer-related mortality in India, and the majority of patients do not receive adequate therapy.^18^ This could be improved by early diagnosis, appropriate treatment, and subsequent second-line therapy if required. The ability to select patients for detection of EGFR mutations in their tumors has improved, thus allowing them to have optimal treatment. Really making an impact on mortality resulting from lung cancer in India requires strong public health measures to control tobacco use. Fortunately, this is now being increasingly recognized by the government and provides hope for the future.

## References

[1] National Centre for Disease Informatics and Research, National Cancer Registry Programme, Indian Council of Medical Research: Three-Year Report of Population Based Cancer Registries, 2009-2011. http://www.icmr.nic.in/ncrp/PBCR_Report%202009_2011/ALL_CONTENT/ALL_PDF/Preliminary_Pages.pdf

[2] Centers for Disease Control and Prevention, Disability and Health Data System: Data Guide, Health Topics: Smoking Status. http://dhds.cdc.gov/guides/healthtopics/indicator?i=smokingstatus

[3] Ventana Medical Systems and Roche Diagnostics International: VENTANA ALK Scoring Interpretation Guide, October 2012, Revision D. http://www.uclad.com/newsletters/ALK-LUNG-IHC-INTERPRETATION-GUIDE.pdf

[4] Therasse P, Arbuck SG, Eisenhauer EA (2000). New guidelines to evaluate the response to treatment in solid tumors: European Organization for Research and Treatment of Cancer, National Cancer Institute of the United States, National Cancer Institute of Canada. J Natl Cancer Inst.

[5] Singh N, Aggarwal AN, Behera D (2012). Management of advanced lung cancer in resource-constrained settings: A perspective from India. Expert Rev Anticancer Ther.

[6] Behera D, Balamugesh T (2004). Lung cancer in India. Indian J Chest Dis Allied Sci.

[7] Rajappa S, Gundeti S, Talluri MR (2008). Chemotherapy for advanced lung cancer: A 5-year experience. Indian J Cancer.

[8] Malik PS, Sharma MC, Mohanti BK (2013). Clinico-pathological profile of lung cancer at AIIMS: A changing paradigm in India. Asian Pac J Cancer Prev.

[9] Bhatt VR, D’Souza SP, Smith LM, et al: Epidermal growth factor receptor mutational status and brain metastases in non–small-cell lung cancer. J Glob Oncol 3:208-217, 2017 10.1200/JGO.2016.003392 PMC5493216 28717762

[10] Rosell R, Carcereny E, Gervais R (2012). Erlotinib versus standard chemotherapy as first-line treatment for European patients with advanced EGFR mutation-positive non-small-cell lung cancer (EURTAC): A multicentre, open-label, randomised phase 3 trial. Lancet Oncol.

[11] Zhou C, Wu YL, Chen G (2011). Erlotinib versus chemotherapy as first-line treatment for patients with advanced EGFR mutation-positive non-small-cell lung cancer (OPTIMAL, CTONG-0802): A multicentre, open-label, randomised, phase 3 study. Lancet Oncol.

[12] Jindal SK, Behera D (1990). Clinical spectrum of primary lung cancer: Review of Chandigarh experience of 10 years. Lung India.

[13] Doval D, Prabhash K, Patil S (2015). Clinical and epidemiological study of EGFR mutations and EML4-ALK fusion genes among Indian patients with adenocarcinoma of the lung. Onco Targets Ther.

[14] Mok TS, Wu YL, Thongprasert S (2009). Gefitinib or carboplatin-paclitaxel in pulmonary adenocarcinoma. N Engl J Med.

[15] Desai SS, Shah AS, Prabhash K (2013). A year of anaplastic large cell kinase testing for lung carcinoma: Pathological and technical perspectives. Indian J Cancer.

[16] McLeer-Florin A, Moro-Sibilot D, Melis A (2012). Dual IHC and FISH testing for ALK gene rearrangement in lung adenocarcinomas in a routine practice: A French study. J Thorac Oncol.

[17] Pandey AV, Phillip DS, Noronha V (2015). Maintenance pemetrexed in nonsmall cell lung carcinoma: Outcome analysis from a tertiary care center. Indian J Med Paediatr Oncol.

[18] Dikshit R, Gupta PC, Ramasundarahettige C (2012). Cancer mortality in India: A nationally representative survey. Lancet.

